# Wavelength dependent mechanism of phenolate photooxidation in aqueous solution†

**DOI:** 10.1039/d3sc00016h

**Published:** 2023-02-20

**Authors:** Kate Robertson, William G. Fortune, Julia A. Davies, Anton N. Boichenko, Michael S. Scholz, Omri Tau, Anastasia V. Bochenkova, Helen H. Fielding

**Affiliations:** a Department of Chemistry, University College London 20 Gordon Street London WC1H 0AJ UK h.h.fielding@ucl.ac.uk; b Department of Chemistry, Lomonosov Moscow State University 119991 Moscow Russia

## Abstract

Phenolate photooxidation is integral to a range of biological processes, yet the mechanism of electron ejection has been disputed. Here, we combine femtosecond transient absorption spectroscopy, liquid-microjet photoelectron spectroscopy and high-level quantum chemistry calculations to investigate the photooxidation dynamics of aqueous phenolate following excitation at a range of wavelengths, from the onset of the S_0_–S_1_ absorption band to the peak of the S_0_–S_2_ band. We find that for *λ* ≥ 266 nm, electron ejection occurs from the S_1_ state into the continuum associated with the contact pair in which the PhO˙ radical is in its ground electronic state. In contrast, we find that for *λ* ≤ 257 nm, electron ejection also occurs into continua associated with contact pairs containing electronically excited PhO˙ radicals and that these contact pairs have faster recombination times than those containing PhO˙ radicals in their ground electronic state.

## Abstract 2

Phenolate photooxidation is integral to a range of biological processes, yet the mechanism of electron ejection has been disputed.

## Graphical abstract

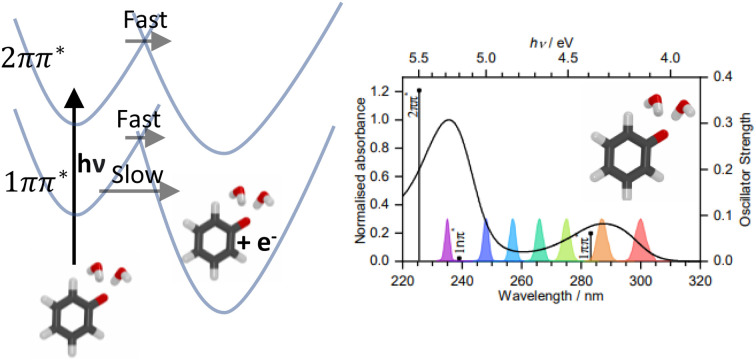

## 1 Introduction

Photooxidation is observed in many biological systems following excitation with ultraviolet (UV) light and, even though it is a driving force for many important photochemical reactions, for example, in the photocycle of fluorescent proteins,^1–3^ it can also have deleterious effects, such as DNA photodamage.^4^ Phenolate has been studied extensively as a model for photooxidation in a range of biological systems, *e.g.*, tyrosine and the green fluorescent protein (GFP) and photoactive yellow protein (PYP) chromophores.^5–10^ Nonetheless, there remains some controversy over the mechanism of phenolate photooxidation.

**Fig. 1:**
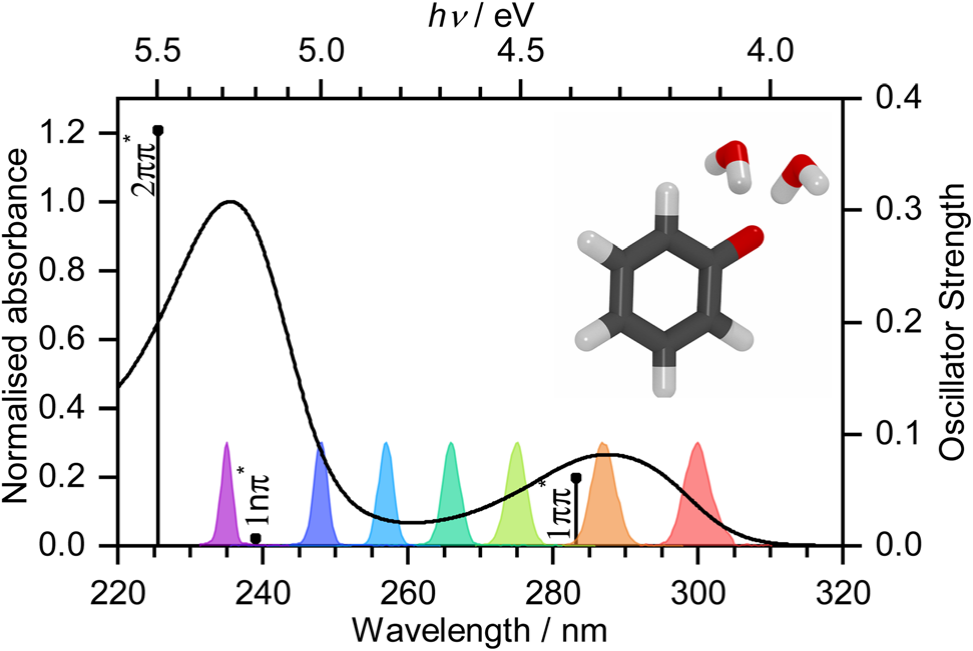
UV-vis spectrum of 20 mM aqueous solution of phenolate. Vertical lines mark XMCQDPT2/SA(7)-CASSCF(10,8)/(aug)-cc-pVDZ//EFP calculated vertical excitation energies (VEEs) with heights proportional to oscillator strengths. Gaussians mark the spectral profiles of the femtosecond pump pulses employed in the work reported here. Inset: PBE0/(aug)-cc-pVDZ//EFP(1043) equilibrium geometry of PhO^−^ + 2H_2_O.

An early picosecond transient absorption spectroscopy (TAS) study found that electron ejection was essentially instantaneous following photoexcitation at 265 nm.^11^ The first detailed femtosecond TAS study, by Chen *et al.*,^12^ found that 266 nm photoexcitation, high in the first absorption band (Fig. 1), generated solvated electrons, e_(aq)_^−^, on a timescale that was longer than typical charge-transfer-to-solvent (CTTS) processes in inorganic anions.^13^ A detailed global target analysis identified two competing pathways for e_(aq)_^−^ formation: fast (sub-picosecond) electron ejection from vibrationally hot S_1_(1ππ*), hereafter termed simply S_1_, and vibrational relaxation followed by slow (few picosecond) electron ejection from vibrationally cold S_1_. A more recent femtosecond TAS study by Tyson and Verlet^14^ found that 257 nm photoexcitation, between the first and second absorption bands (Fig. 1), generated e_(aq)_^−^ on a fast, sub-picosecond, timescale that was comparable with typical charge-transfer-to-solvent (CTTS) processes in inorganic anions. They concluded that the fast electron ejection was from vibrationally hot S_1_ and, to explain the difference between their observations and those of Chen *et al.*,^12^ proposed a Marcus model in which e_(aq)_^−^ were formed *via* a tunnelling mechanism. We were curious as to why electron detachment from higher lying S_1n_(1nπ*) and S_2_(2ππ*) states, hereafter termed simply S_1n_ and S_2_, had not been considered. This led us to undertake a systematic femtosecond TAS study of the photooxidation dynamics of aqueous phenolate following photoexcitation at a range of wavelengths, from the onset of the S_0_–S_1_ absorption band to the peak of the S_0_–S_2_ band, complemented by liquid-microjet photoelectron spectroscopy (LJ-PES) measurements and high-level quantum chemistry calculations.

## 2 Experimental and computational methods

### 2.1 Experimental methods

Phenol was purchased (≥99%, Sigma-Aldrich) and used without further purification. Steady state UV-visible absorption spectra of aqueous phenolate were recorded using a PerkinElmer LAMBDA 365 spectrophotometer. Femtosecond laser spectroscopy experiments employed laser pulses derived from a regenerative amplifier seeded by a Ti:sapphire oscillator (Coherent Astrella-HE-USP). Tuneable laser pulses were obtained using optical parametric amplifiers (OPAs; Coherent OPerA Solo).

The TA spectra were recorded using a commercial transient absorption spectrometer (Ultrafast Systems Helios Fire) following photoexcitation at seven pump wavelengths over the range 300–235 nm (4.13–5.28 eV). The UV pump beam was generated from one OPA and was attenuated to achieve a pulse energy of around 100 nJ at the sample. The broadband probe beam was created *via* white-light generation by focussing the 800 nm fundamental beam into a sapphire (visible) or calcium fluoride (UV) plate, giving a combined probe range spanning 350–720 nm. The relative polarisations of the pump and probe beam were set at the magic angle 54.7° to suppress polarisation effects. Solutions of 20 mM aqueous phenolate were prepared from aqueous phenol with concentrated sodium hydroxide solution added to achieve pH 13. The phenolate sample was flowed continuously at 10 mL min^−1^ through a Harrick cell with a 100 μm path length using a liquid diaphragm pump (KNF, Simdos 02). The instrument response function was determined at each pump wavelength using a fit to solvent-only spectra (Fig. S1†).

Photoelectron spectra of aqueous phenolate were recorded using our liquid microjet photoelectron spectrometer that has been described in detail elsewhere.^15^ Solutions of aqueous phenolate were prepared by addition of 0.1 mM phenol to ∼2 mM NaOH solution; all solutions were made up with pure water (resistivity >15 MΩ cm^−1^). The NaOH concentration was adjusted slightly to flatten the potential in the interaction region of the spectrometer,^16^ with complete formation of the phenolate anion verified by UV-visible absorption spectroscopy. Aqueous phenolate solution was passed into the spectrometer by a high-performance liquid-chromatography pump, operating with backing pressures ∼70 bar. The resulting microjet had a nominal diameter of 20 μm at a flow rate of 0.7 mL min^−1^. The solution was intersected with femtosecond pulses of ultraviolet light around 2 mm downstream from the liquid microjet nozzle and the resulting photoelectrons were guided by a strong inhomogeneous magnetic field into the time-of-flight region. The interaction region was around 1 mm from the skimmer, and the polarisation of the laser light was parallel to the time-of-flight axis. Photoelectron spectra of NO and Xe were recorded to measure the instrument function, potential gradient in the interaction region, and the vacuum level offset between the interaction region and the analyser.^1,17^

### 2.2 Computational details

The structure of aqueous phenolate was constructed as a single molecule placed at the centre of a large box of water molecules with a length of 100 Å. Initially, molecular dynamics (MD) simulations were performed with NAMD.^18^ The CHARMM force field parameters were used to describe the solute,^19^ and the water molecules were described with the modified TIP3P parameters.^20,21^ The system was optimised for 5000 steps and MD simulations were carried out with an integration step of 1 fs for 2 ns. Periodic boundary conditions were applied during room-temperature simulations using the *NVT* ensemble. The system was gradually cooled down to 20 K in steps of 1 K during 560 ps to get a representative lowest-energy minimum on the multidimensional ground-state potential energy surface. The final MD geometry was obtained by performing geometry optimization for an additional 10 000 steps. The structure was then reduced in size by cutting it into a sphere of a radius of 17 Å with ∼1000 water molecules for subsequent quantum chemistry calculations coupled to the Effective Fragment Potential method (EFP).^22^ The quantum (QM) part included phenolate and two water molecules, which were hydrogen-bonded to the chromophore, while all other water molecules were treated as EFPs. The structure was optimized using the PBE0 (ref. 23 and 24)/(aug)-cc-pVDZ^25^//EFP method with diffuse functions affixed to the oxygen atoms. The optimized structure was used for calculating vertical excitation energies (VEEs) in the phenolate anion and its radical in their ground electronic states at the equilibrium geometry of the anion as well as their transient absorption from the S_1_, D_0_, and D_1_ states.

The VEEs of aqueous phenolate were calculated using the extended multi-configuration quasi-degenerate perturbation theory, XMCQDPT2,^26,27^ combined with the EFP method. The zeroth-order wave functions were constructed using the state-averaged complete active space self-consistent field method, SA-CASSCF. The active space included all valence π and π* orbitals as well as the n-orbital located at the oxygen atom. The XMCQDPT2 calculations employed a one-particle DFT/PBE0-based Fock matrix to obtain energies of all CASSCF semi-canonical orbitals.

The optimized structure of PhO^−^ + 2H_2_O inside the water sphere of 1043 EFPs was also used in large-scale hybrid calculations of the first vertical detachment energy (D_0_ VDE) of aqueous phenolate. A series of structures of increasing size with additional outer-shell water molecules was constructed to test convergence of the calculated VDE with respect to the system size. The fully optimized QM/EFP system was additionally solvated with a large box of water molecules with a length of 100 Å. MD simulations with periodic boundary conditions were performed with the QM/EFP core kept frozen. The same MD protocol was used as in the initial MD step. Following equilibration, the system was slowly cooled down and then optimized. The final structure was cut into smaller-sized systems, with distances from water molecules to the chromophore ranging from 10 Å to 40 Å. The VDEs were calculated at the PBE0/(aug)-cc-pVDZ//EFP level of theory as a difference in energy between the chromophore anion and its neutral radical. Both the inner and outer water shells were treated as EFPs in these calculations. The calculated VDE reached a converged value of 7.3 eV for a system with ∼11 250 water molecules. This value was further validated by high-level XMCQDPT2/SA(10)-CASSCF(8,8)/(aug)-cc-pVDZ+//EFP calculations, with both detached and valence states of the anion being treated simultaneously using a state-averaging procedure. The computational details are described elsewhere.^1^ All XMCQDPT2/EFP calculations were performed omitting the fragment polarization term, thus only allowing the QM electron density to be polarized in the field of effective fragments. The solvent response was calculated separately in a fully self-consistent manner at the PBE0/EFP level of theory and used as a correction to the XMCQDPT2/EFP results.

The electronic structure theory calculations were performed within the Firefly computational package,^27^ which is partially based on the GAMESS (US) source code.^28^

## 3 Results and discussion

**Fig. 2:**
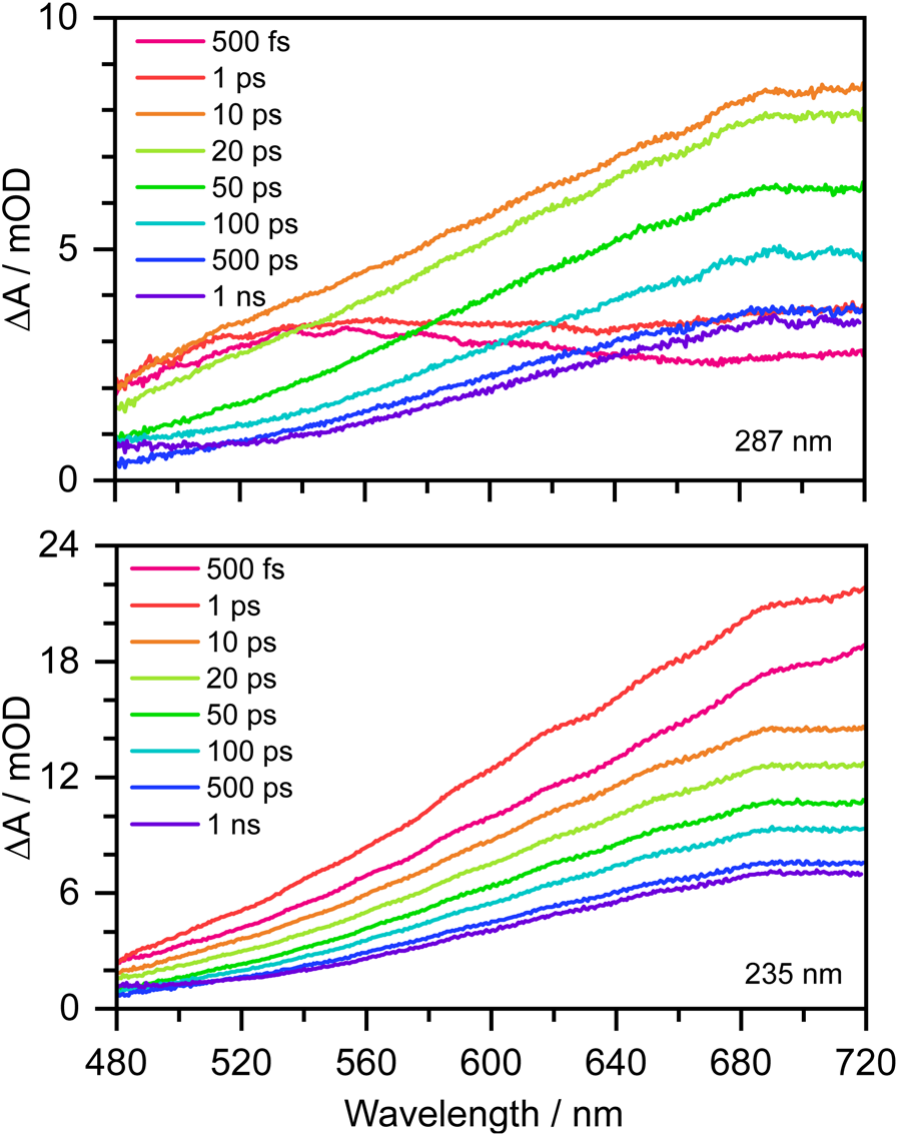
480–720 nm transient absorption spectra of 20 mM aqueous phenolate at displayed pump-probe delays following photoexcitation at 287 nm (top) and 235 nm (bottom). The broad absorption centered around 700 nm is attributed to solvated electrons and the absorption feature with a peak around 540 nm is attributed to the S_1_ state of phenolate.^12^

Transient absorption maps of 20 mM aqueous phenolate following photoexcitation at seven pump wavelengths in the range 300–235 nm are presented in Fig. S2–S8.†Fig. 2 shows the transient absorption spectra at different pump-probe delays following photoexcitation at the maxima of the S_0_–S_1_ and S_0_–S_2_ absorption bands, at 287 nm and 235 nm, respectively. Following photoexcitation of S_1_ at 287 nm, an absorption band centered around 540 nm appears, effectively instantaneously. The spectral profile changes substantially between 1 ps and 10 ps to reveal a broad absorption band centered near 700 nm, which decreases in intensity between 10 ps and 1 ns without significantly changing its spectral profile. From the work of Chen *et al.*,^12^ we can assign the feature around 540 nm to an excited state absorption (ESA) of the phenolate S_1_ state, and the broad absorption around 700 nm is characteristic of e_(aq)_^−^. Our XMCQDPT2/SA(15)-CASSCF(10,8)/(aug)-cc-pVDZ//EFP calculations support this assignment. In the visible region, the most bright vertical transition from S_1_ is located at 547 nm with an oscillator strength of 0.07 and corresponds to S_3_ excitation. In contrast, following photoexcitation of S_2_ at 235 nm, the e_(aq)_^−^ absorption spectrum appears effectively instantaneously and starts decreasing rapidly after around 1 ps. There is no evidence of S_1_ ESA.

**Fig. 3:**
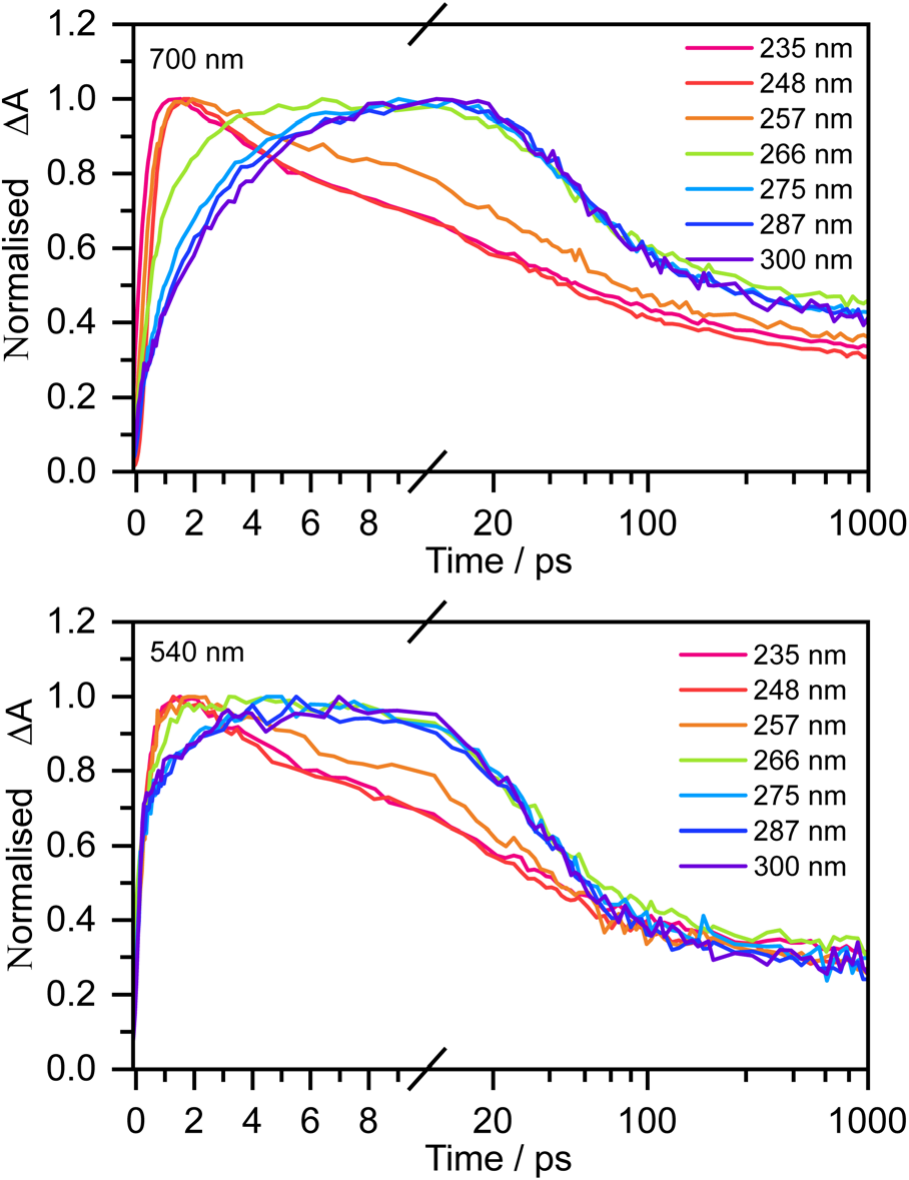
Kinetic traces of the transient absorption spectra of 20 mM aqueous phenolate at a probe wavelength of 700 nm (top) and 540 nm (bottom) and displayed pump wavelengths. Data have been normalised to maximum Δ*A*.

Fig. 3 shows normalised kinetic traces of 20 mM aqueous phenolate at 540 nm (the S_1_ ESA) and 700 nm (e_(aq)_^−^ absorption) following photoexcitation at seven wavelengths, from the origin of the S_0_–S_1_ absorption band at 300 nm, to the peak of the S_0_–S_2_ band at 235 nm, including the wavelengths employed in the earlier femtosecond TAS studies (266 nm and 257 nm).^12,14^ Kinetic traces before normalisation are presented in Fig. S9.† Even though the spectral profiles associated with S_1_ and e_(aq)_^−^ extend over the full probe range (350–720 nm), their relative contributions to the transient absorption spectra can be deconvoluted due to their substantially different spectral profiles.

The 700 nm kinetic traces show that e_(aq)_^−^ formation is more rapid following photoexcitation of S_2_ (*λ* ≤ 248 nm) compared to photoexcitation of S_1_ (*λ* ≥ 266 nm). It is also clear that there are two distinct timescales for e_(aq)_^−^ formation following photoexcitation of S_1_: an initial few picosecond rise followed by a slower approximately 20 ps rise matching the fluorescence lifetime of S_1_ recorded by Chen *et al.*^12^ Interestingly, the timescales for e_(aq)_^−^ decay also appear to depend on the photoexcitation wavelength: following photoexcitation of S_2_, the e_(aq)_^−^ absorbance begins to decay within a few picoseconds, whereas following photoexcitation of S_1_, the absorbance only begins to decay after around 10 ps.

The 540 nm kinetic traces are also dependent on photoexcitation wavelength. Following photoexcitation with *λ* ≤ 248 nm, the 540 nm kinetic traces are virtually identical to the 700 nm kinetic traces. In contrast, following photoexcitation of S_1_ (*λ* ≥ 266 nm), the 540 nm transient absorption appears effectively instantaneously, then after a few picoseconds it begins to resemble the 700 nm transient absorption.

For 257 nm photoexcitation, it is clear from the 700 nm kinetic trace that there is a slower decay component in the 2–20 ps region that is characteristic of the S_1_ relaxation dynamics, and that the long time dynamics (100–1000 ps) has a contribution from the slower decay characteristic of S_1_. The contribution of S_1_ is confirmed by comparing kinetic traces at probe wavelengths of 515 and 680 nm (Fig. S16†).

Time constants obtained from fits to the transient absorption spectra at the six pump wavelengths corresponding to photoexcitation of either S_1_ (*λ* ≥ 266 nm) or S_2_ (*λ* ≤ 248 nm) are listed in Table 1. Following photoexcitation of S_1_, the spectra were fit using a procedure based on that employed by Chen *et al.*^12^ Following photoexcitation of S_2_, the spectra were best fit with four exponentials (one rise and three decays). Both fitting procedures are described in the ESI (Fig. S10–S16†). Photoexcitation at 257 nm is more complex and will be discussed later.

**Table 1:** Table of time constants (in ps) obtained from fits to the TAS spectra (Fig. S2–S7). *τ*_instr_ is the instrument response function; an example fit can be found in the ESI. 
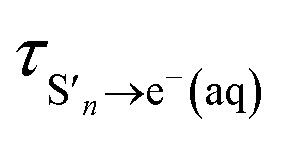
 is the timescale of e_(aq)_^−^ formation from directly populated ‘hot’ S_1_ or S_2_ and *τ*_S_1_→e^−^(aq)_ is the timescale of e_(aq)_^−^ formation from ‘cold’ S_1_. *τ*_r1_, *τ*_r2_ and *τ*_r3_ describe the decay of e_(aq)_^−^ by geminate recombination

*λ*_pump_/nm	*τ*_instr_	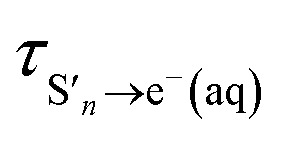	*τ*_S_1_→e^−^(aq)_	*τ*_r1_	*τ*_r2_	*τ*_r3_
235	0.4	0.4 ± 0.2		6.6 ± 0.4	99 ± 9	>1000
248	0.4	0.4 ± 0.3		6.2 ± 0.5	90 ± 8	>1000
266	0.4	1.02 ± 0.03	25 ± 1	36 ± 6	210 ± 50	
275	0.4	2.3 ± 0.1	23 ± 0.8	54 ± 5	550 ± 300	
287	0.3	2.6 ± 0.1	23 ± 1	50 ± 3	490 ± 200	
300	0.3	3.5 ± 0.1	21 ± 0.8	52 ± 4	>1000	

The time constants show that following photoexcitation to S_1_ (*λ* ≥ 266 nm), solvated electrons are formed on both few-picosecond and ∼20 ps timescales (
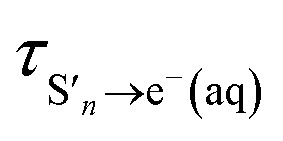
 and *τ*_S_1_→e^−^(aq)_, respectively). This is consistent with the earlier assignment of two timescales to fast electron ejection from vibrationally hot S_1_, and vibrational relaxation followed by slower electron ejection from vibrationally cold S_1_.^12^ Following photoexcitation at 300 nm, close to the bottom of the well, the slow electron emission occurs on a timescale of 21 ± 0.8 ps which is very similar to the fluorescence lifetime of S_1_ measured by Chen *et al.*^12^ (22 ± 2 ps). In contrast, following photoexcitation to S_2_ (*λ* ≤ 248 nm), solvated electrons are formed on a sub-picosecond timescale and there is no evidence of the slower electron formation associated with relaxation to vibrationally cold S_1_. A similar observation was reported by Tyson and Verlet following photoexcitation at 257 nm.^14^

Following photoexcitation to S_1_ with *λ* in the range 287–266 nm, the e_(aq)_^−^ decays are best described by fitting with two exponential decays, *τ*_r1_ and *τ*_r2_. Although geminate recombination is a complex process, and fitting exponentials only estimates the timescales, the *τ*_r1_ timescales obtained from our fits are of the same order of magnitude as the faster recombination timescale observed in the earlier 266 nm TAS measurement (50 ps).^12^ It is worth noting that the e_(aq)_^−^ absorbance does not decay to zero within our experimental window of 1 ns. Interestingly, we found that following photoexcitation at 300 nm, *τ*_r2_ > 1000 ps, which is longer than our experimental window and thus has a large associated error (ESI†). Following photoexcitation to S_2_ (248 and 235 nm), the e_(aq)_^−^ decays are best described by fitting with three exponential decays, *τ*_r1_, *τ*_r2_ and *τ*_r3_. The last of these has a timescale longer than that of our experimental window of 1 ns. *τ*_r1_ and *τ*_r2_ are noticeably shorter than the equivalent timescales observed following photoexcitation of S_1_. This suggests that the PhO˙ radical could be formed in a higher lying electronically excited state, where recombination would be expected to be faster since the corresponding electron would have less kinetic energy, following photoexcitation at 248 and 235 nm compared to photoexcitation at *λ* ≥ 266 nm.

It is interesting to note that the fast time constant, 
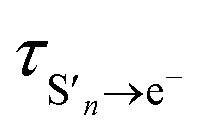
, decreases following S_1_ photoexcitation from 300–266 nm. The decrease is most marked between 275 and 266 nm, which could suggest that there is some contribution from the S_2_ state at 266 nm. However, the long time dynamics (100–1000 ps) in the 700 nm kinetic trace for 266 nm photoexcitation follows that of S_1_ and there is no noticeable contribution from S_2_. Therefore, we attribute this decrease in the fast time constant to increasing vibrational energy in S_1_. In the Marcus picture,^14^ the tunnelling rate from S_1_ to the contact pair is very sensitive to barrier height and width, so the marked decrease in fast time constant between 275 and 266 nm photoexcitation could be because 266 nm excites close to the top of the barrier.

At 257 nm, simultaneous photoexcitation of S_1_ and S_2_ makes it difficult to separate the contributions from the S_1_ and S_2_ photooxidation mechanisms. The presence of the S_1_ ESA (Fig. S16†) as well as the faster electron formation time (consistent with the 257 nm TAS measurements reported by Tyson and Verlet^14^) and faster electron recombination time, confirm that 257 nm photoexcitation populates both S_1_ and S_2_.

**Fig. 4:**
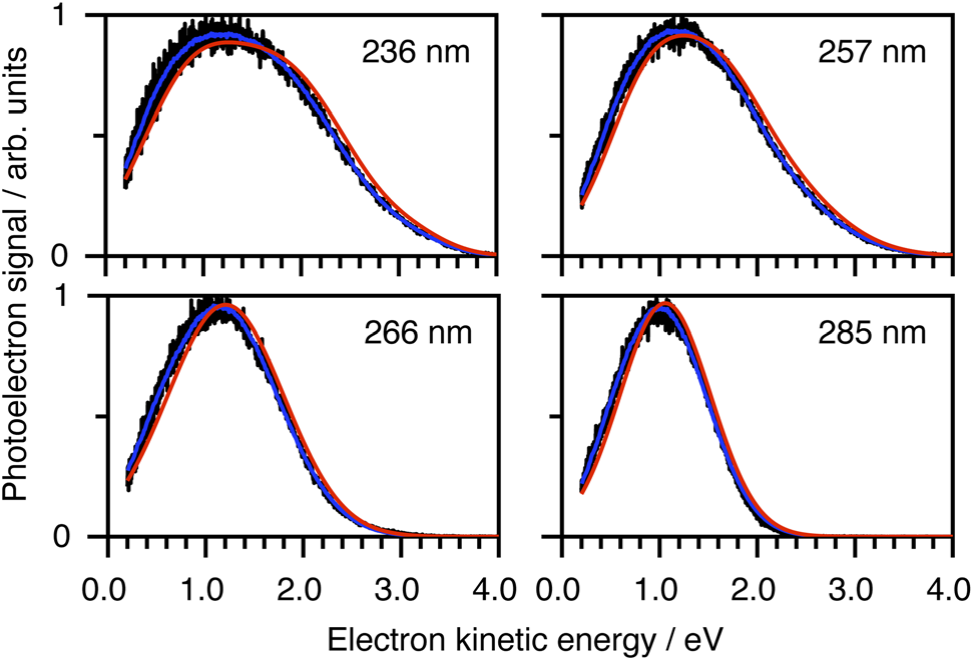
Resonance-enhanced 1 + 1 photoelectron spectra (PES) of aqueous phenolate at 236, 257, 266 and 285 nm (black) together with retrieved true PES (solid red lines).^31^ The 285 nm spectrum is adapted according to CC-BY license from ref. 31. Copyright 2022 the authors. Published by American Chemical Society under a Creative Commons CC BY 4.0 license.

To determine which photodetachment continua are accessible from the different electronically excited states of phenolate accessed in our measurements, we recorded photoelectron spectra following 1 + 1 resonance-enhanced photodetachment of aqueous phenolate at 285 nm (*via* S_1_), 236 nm (*via* S_2_), and 266 and 257 nm (wavelengths employed in the earlier TAS studies^12,14^). The spectra are plotted as a function of electron kinetic energy (eKE) in Fig. 4. The measured spectra are slightly distorted as a result of inelastic scattering of the photoelectrons in liquid water,^16,17,29,30^ so the true photoelectron spectra were retrieved using the algorithm described in ref. 31 and are overlaid on the measured spectra, in red. The photoelectron spectra obtained following photoexcitation at 285 nm (close to the peak of the S_0_–S_1_ absorption band) and 266 nm, correspond to resonance-enhanced detachment *via* S_1_ and are best fit by single Gaussians. In contrast, the broader spectra obtained following photoexcitation at 236 nm (close to the peak of the S_0_–S_2_ absorption band) and 257 nm, are best fit by three Gaussians (Fig. S17 and Table S1†). The increased broadening and number of Gaussians required to fit the two shorter wavelength photoelectron spectra compared to the two longer wavelength photoelectron spectra, indicates that higher lying electronically excited states of phenolate are accessible at 257 nm as well as 236 nm, and that these may be coupled to solvated electron continua associated with higher lying electronic states of the PhO˙ radical, supporting our interpretation of the different timescales for geminate recombination observed in our femtosecond TAS measurements.

**Fig. 5:**
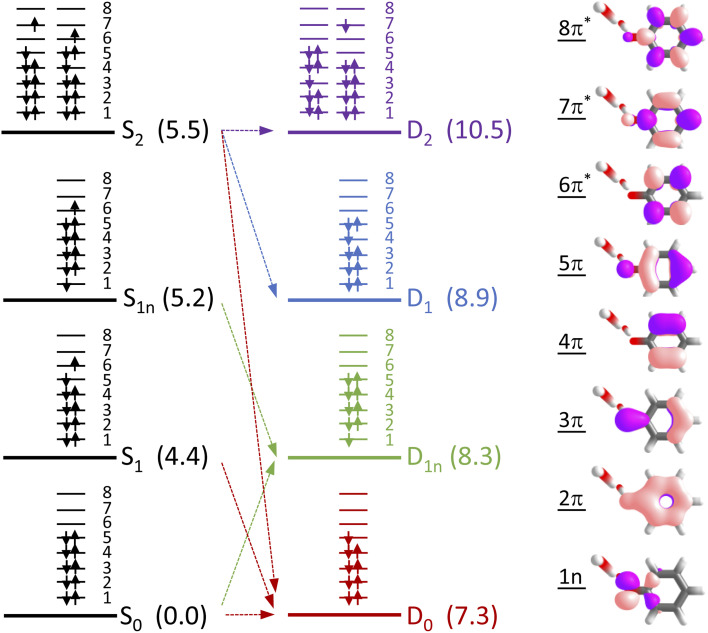
Electronic configurations of the four lowest singlet electronic states of phenolate and four lowest doublet electronic states of the corresponding radical in aqueous solution. Numbers in brackets refer to XMCQDPT2/SA(7)-CASSCF(10,8)/(aug)-cc-pVDZ//EFP and XMCQDPT2/SA(7)-CASSCF(9,8)/(aug)-cc-pVDZ//EFP calculated vertical excitation and vertical detachment energies in eV, relative to S_0_. Arrows highlight possible electron detachment processes. Also shown are the SA(7)-CASSCF(10,8) natural orbitals.

To determine which resonance-enhanced photodetachment pathways are accessible energetically, we carried out high-level quantum chemistry calculations of the electronic structure of the singlet states of PhO^−^ and doublet states of PhO˙ (Fig. 5). Using Koopmans' arguments, we are able to determine that S_0_ is most likely to detach to D_0_ and D_1n_, S_1_ to D_0_, S_1n_ to D_1n_, and S_2_ to D_0_, D_1_ and D_2_. Thus, it is clear that the peaks which are modelled well with single Gaussian functions in the 285 and 266 nm photoelectron spectra correspond to S_1_–D_0_ detachment. The situation is more complicated for the 236 and 257 nm photoelectron spectra, as their lineshapes could only be satisfactorily reproduced using a linear combination of Gaussian functions. The high eKE tails of both spectra lie at similar two-photon binding energies close to the D_0_ binding energy, suggesting that all four spectra contain contributions from detachment to D_0_. The 236 nm photoelectron spectrum likely has contributions from resonance-enhanced photodetachment *via* S_2_ to D_0_, D_1_ and D_2_, although we cannot rule out ultrafast internal conversion to S_1_ followed by photodetachment to D_1_. The 257 nm photoelectron spectrum likely has contributions from excitation of both S_1_ and S_2_, as discussed above. A detailed analysis of the photoelectron spectra of aqueous phenolate at a wide range of photon energies will be reported in a future publication.

**Fig. 6:**
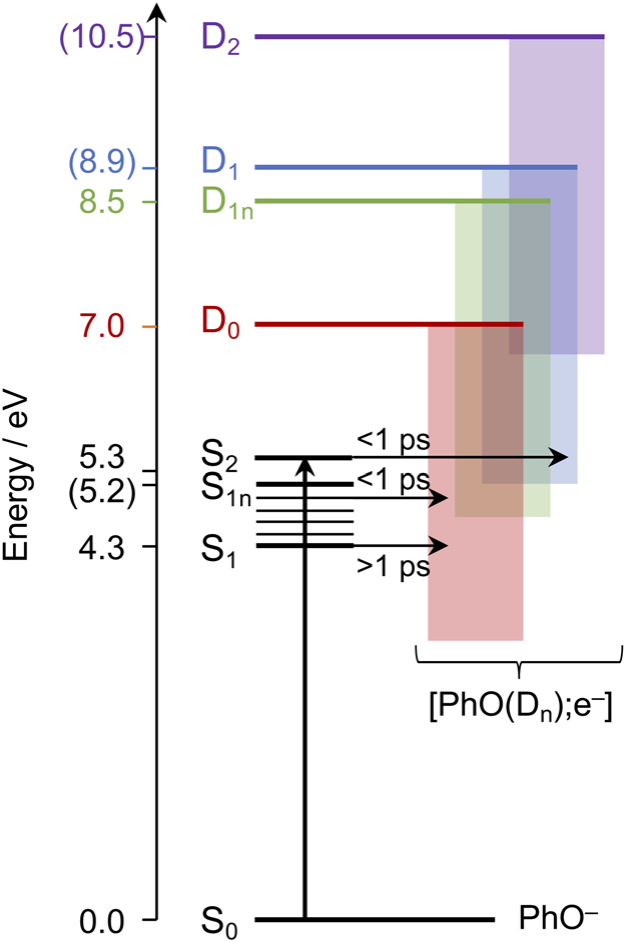
Schematic energy level diagram illustrating the different photooxidation pathways and timescales following photoexcitation of the S_1_, S_1n_ and S_2_ electronically excited states of phenolate in aqueous solution. Numbers not in parentheses are *λ*_max_ (in eV) from the UV-vis spectrum presented in Fig. 1 and VDEs obtained from UV (D_0_) and X-ray (D_1n_) liquid-jet photoelectron spectroscopy measurements.^31,33^ Numbers in parentheses are the calculated VEEs and VDEs presented in Fig. 5. Horizontal arrows represent coupling of S_2_, hot S_1_ and cold S_1_ to the different solvated electron continua (shaded blocks) with representative timescales (see text).

Having explored which photodetachment pathways are accessible from the different electronically excited states, and knowing that the binding energy of the solvated electron is between 3.7 and 3.8 eV,^29,32^ we can explore which photooxidation pathways might be accessible (Fig. 6). The S_1_ state is presumably coupled to the solvated electron continuum associated with the PhO˙ radical in the D_0_ state, and the S_2_ state is likely coupled with the continua associated with the PhO˙ radical in the D_0_ and D_1_ states. Although Tyson and Verlet suggested that it was not possible to access a higher lying detachment continuum following photoexcitation at 257 nm, based on our calculations and the second vertical detachment energy of phenolate reported in ref. 33, we find that it is possible to access the D_1_ continuum from S_2_ following 257 nm photoexcitation. Thus, having determined the possible photooxidation pathways, and observed that higher lying detachment continua may be accessible following 1 + 1 photodetachment at 257 and 236 nm, we conclude that the faster timescale observed for electron recombination following photoexcitation at *λ* ≤ 257 nm could be the result of the PhO˙ radical being formed in an excited electronic state.

**Fig. 7:**
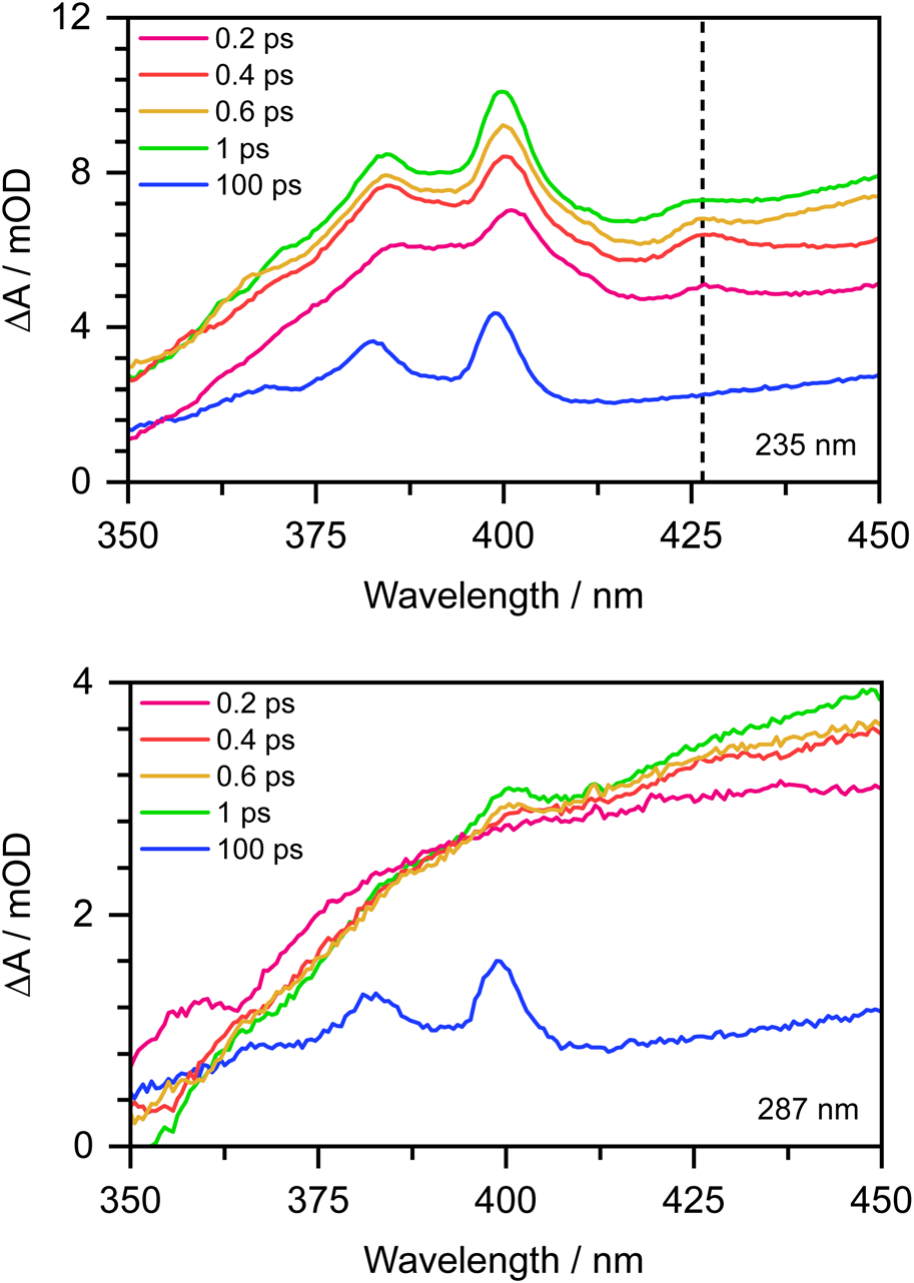
350–450 nm transient absorption spectra of 20 mM aqueous phenolate at displayed pump-probe delays following photoexcitation at 235 nm (top) and 287 nm (bottom). The dashed vertical highlights the peak at 427 nm described in the main text.

To confirm this, as Chen *et al.* observed the formation of the PhO˙ radical in its D_0_ state between 330 and 400 nm,^12^ we recorded the 350–450 nm transient absorption spectrum of aqueous phenolate following photoexcitation at 235 nm to see whether we could find direct evidence of the formation of the PhO˙ radical in a higher lying state (Fig. 7). In addition to the structured band system between 350 and 400 nm reported by Chen *et al.*, we observe an additional peak around 427 nm and a shoulder on the longer wavelength edge of the peak around 400 nm. These additional features decay on a subpicosecond timescale and are not observed in the 350–450 nm transient absorption spectrum of aqueous phenolate following photoexcitation at 287 nm (Fig. 7). Similar features were observed for aqueous phenol following excitation at 200 nm and the small peak at 427 nm was assigned to the phenol radical cation.^34^ Such an assignment would not be consistent with our measurements as they were carried out at pH 13. Instead, we attribute the weak transient absorption around 427 nm to absorption of an excited electronic state of the PhO˙ radical, slightly red-shifted from the absorption band attributed to the radical in its ground electronic state. This is supported by our XMCQDPT2/SA(15)-CASSCF(9,8)/(aug)-cc-pVDZ//EFP calculations which show that in this region, the most bright D_1_ → D_3_ vertical transition is at 411 nm with an oscillator strength of 0.06, red-shifted from the most bright D_0_ → D_2_ transition located at 386 nm with an oscillator strength of 0.1. This is consistent with the earlier observation of this feature, as photolysis of phenol at 200 nm could generate phenoxy radicals in the D_1_ state.^34^ The shoulder on the long wavelength side of the 400 nm feature may arise from vibrational structure associated with the 427 nm feature or to hot/combination bands of the radical ground-state absorption. These UV TAS spectra reinforce our conclusion that photoexcitation at shorter wavelengths generates PhO˙ radicals in higher lying states that undergo more rapid geminate recombination than the PhO˙ radical in its ground electronic state.

## 4 Conclusions

In summary, by using a combination of femtosecond TAS, liquid-microjet photoelectron spectroscopy and quantum chemistry calculations, we have unravelled the wavelength dependant mechanism for phenolate photooxidation. We find that for *λ* ≥ 266 nm, electron ejection occurs from the S_1_ state into the continuum associated with the [PhO˙(D_0_); e^−^] contact pair, with timescales that support the competing pathway mechanism proposed by Chen *et al.*^12^ For *λ* ≤ 248 nm, electron ejection occurs from a higher lying electronically excited state into continua associated with contact pairs in which PhO˙ radicals are also formed in electronically excited states. These contact pairs have faster recombination timescales than the [PhO˙(D_0_); e^−^] contact pair. We found that photoexcitation at 257 nm populated both S_1_ and S_2_, and we propose that the fast electron ejection process previously attributed to tunnelling^14^ actually arises from S_2_.

Understanding the mechanism of phenolate photooxidation following photoexcitation of higher lying excited electronic states is directly relevant to the photooxidation of protein chromophores since the higher lying electronically excited states of both GFP and PYP chromophores have been implicated in the formation of solvated electrons.^1,35–37^ To the best of our knowledge this work represents one of the first direct observations of photooxidation of an organic chromophore in aqueous solution yielding an oxidised species in an electronically-excited state. The subsequent relaxation dynamics of such electronically-excited oxidised species represent an enticing target for future studies, particularly since chromophores containing phenolate motifs are ubiquitous in biology.

## Author contributions

The project was conceived and supervised by H. H. F. The TAS data were recorded by J. A. D., with assistance from K. R., and processed, analysed and interpreted by K. R., with contributions from J. A. D. and M. S. The PES data were recorded by W. G. F., with assistance from O. T., and processed, analysed and interpreted by W. G. F., with assistance from M. S. Calculations were undertaken and interpreted by A. N. B. and A. V. B. The manuscript was written by K. R. and H. H. F. with contributions from A. V. B., W. G. F., M. S. and J. A. D. All authors read and approved the final version of the manuscript.

## Conflicts of interest

There are no conflicts to declare.

## Supplementary Material

SC-014-D3SC00016H-s001
