# Supplementary material for: Wavelength dependent mechanism of phenolate photooxidation in aqueous solution
Source: Chem Sci. 2023 Feb 20;14(12):3257–64. doi: 10.1039/d3sc00016h (PMC10034221; doi:10.1039/d3sc00016h)
Supplement: SC-014-D3SC00016H-s001 [file SC-014-D3SC00016H-s001.pdf]

Electronic Supplementary Material (ESI) for Chemical Science.
This journal is © The Royal Society of Chemistry 2023

Supplementary Information

Wavelength dependent mechanism of phenolate photooxidation in aqueous solution

Kate Robertson,† William G. Fortune,† Julia A. Davies,† Anton N. Boichenko,‡ Michael S. Scholz,† Omri Tau,† Anastasia V. Bochenkova,‡ and Helen H. Fielding[1]†

†*Department of Chemistry, University College London, 20 Gordon Street, London, WC1H 0AJ, U.K.*

‡*Department of Chemistry, Lomonosov Moscow State University, 119991 Moscow, Russia*

E-mail: h.h.fielding@ucl.ac.uk

[1] To whom correspondence should be addressed

S1 Transient absorption spectroscopy

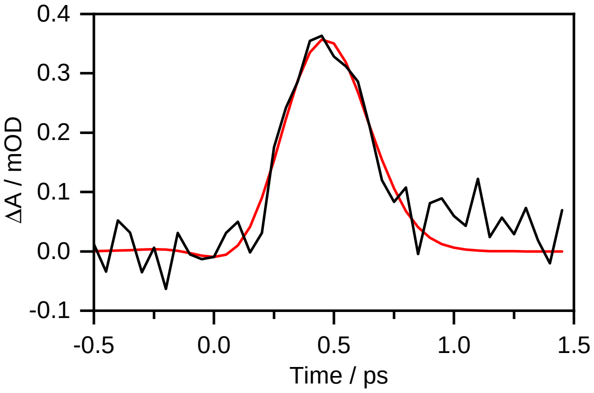

Figure S1: Exemplar fit (red) to determine the instrument response function (IRF) using Surface Xplorer software for pump and probe wavelengths of 235 nm and 520 nm, respectively. The full width half maximum (FWHM) of the fit (red) provides the IRF, which in this case is 490 fs. For each pump wavelength, this process is completed at several probe wavelengths and averaged.

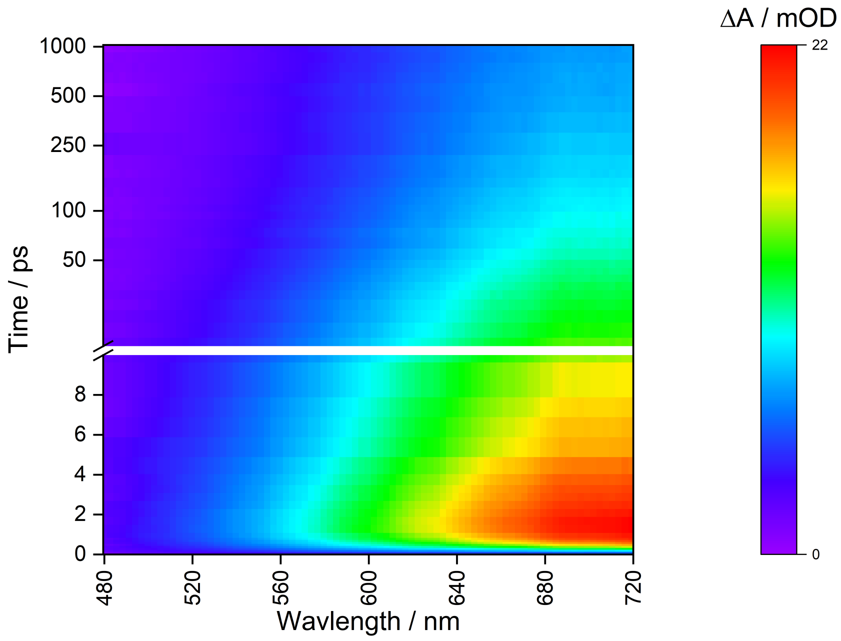

Figure S2: Heat-map of the full 2D transient absorption data set of 20 mM phenolate following photoexcitation at 235 nm. The time axis has a linear scale from 0-10 ps and a logarithmic from 10-1000 ps.

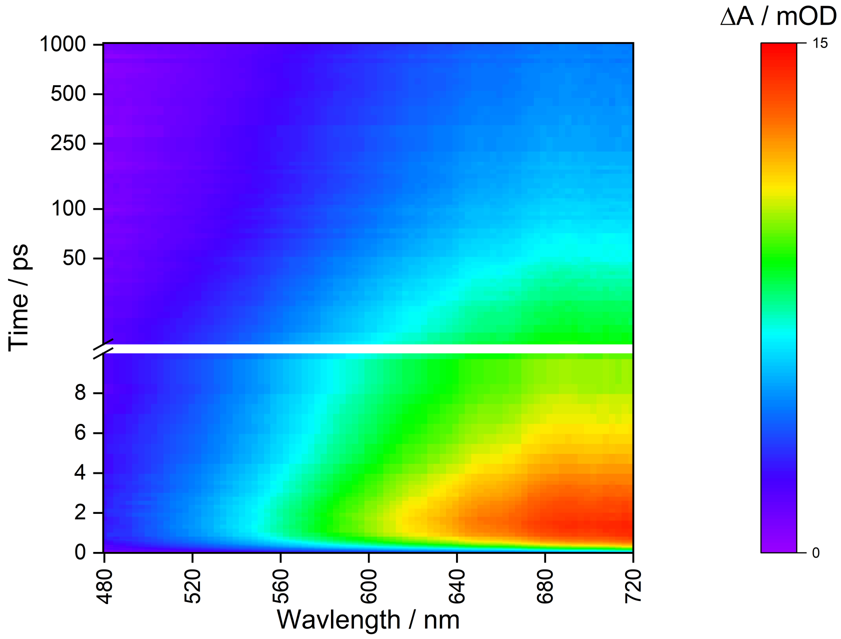

Figure S3: Heat-map of the full 2D transient absorption data set of 20 mM phenolate following photoexcitation at 248 nm. The time axis has a linear scale from 0-10 ps and a logarithmic from 10-1000 ps.

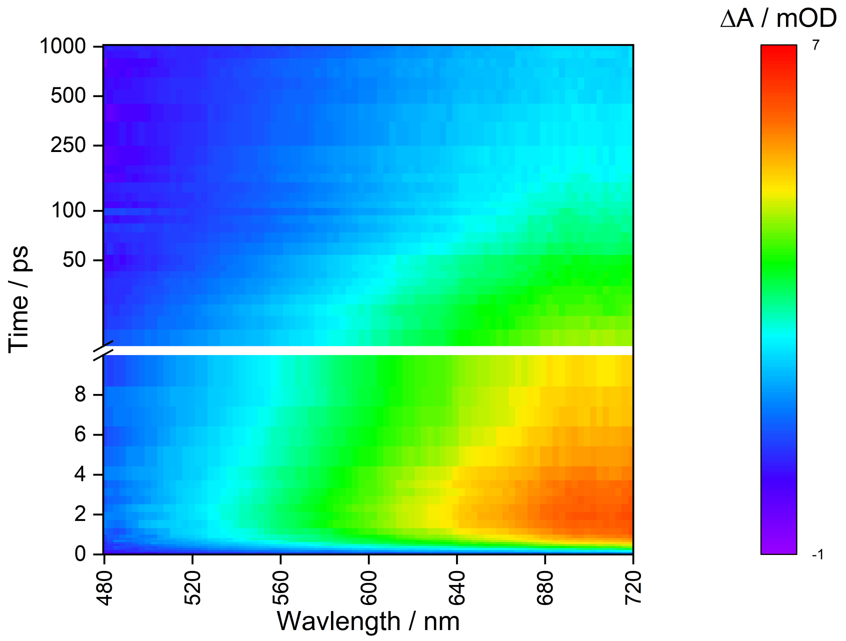

Figure S4: Heat-map of the full 2D transient absorption data set of 20 mM phenolate following photoexcitation at 257 nm. The time axis has a linear scale from 0-10 ps and a logarithmic from 10-1000 ps.

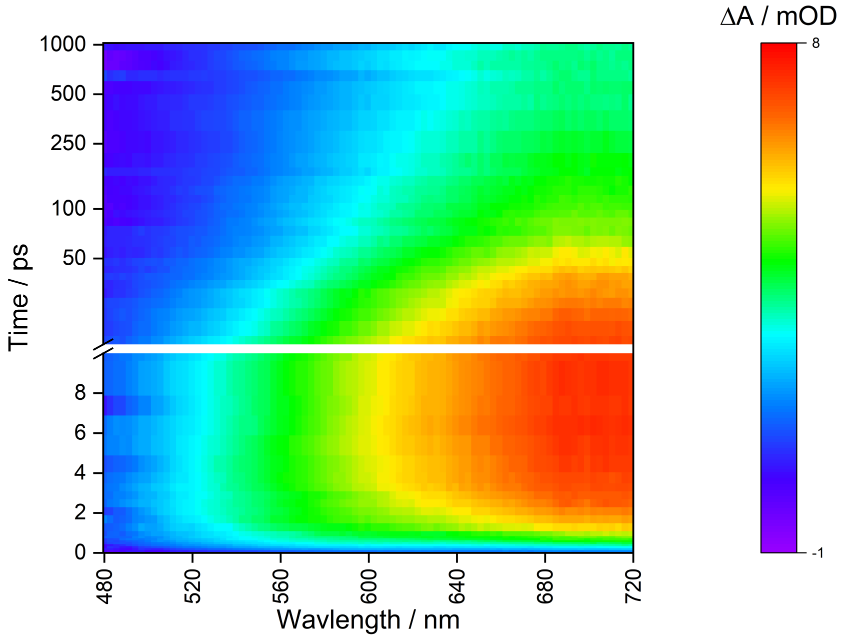

Figure S5: Heat-map of the full 2D transient absorption data set of 20 mM phenolate following photoexcitation at 266 nm. The time axis has a linear scale from 0-10 ps and a logarithmic from 10-1000 ps.

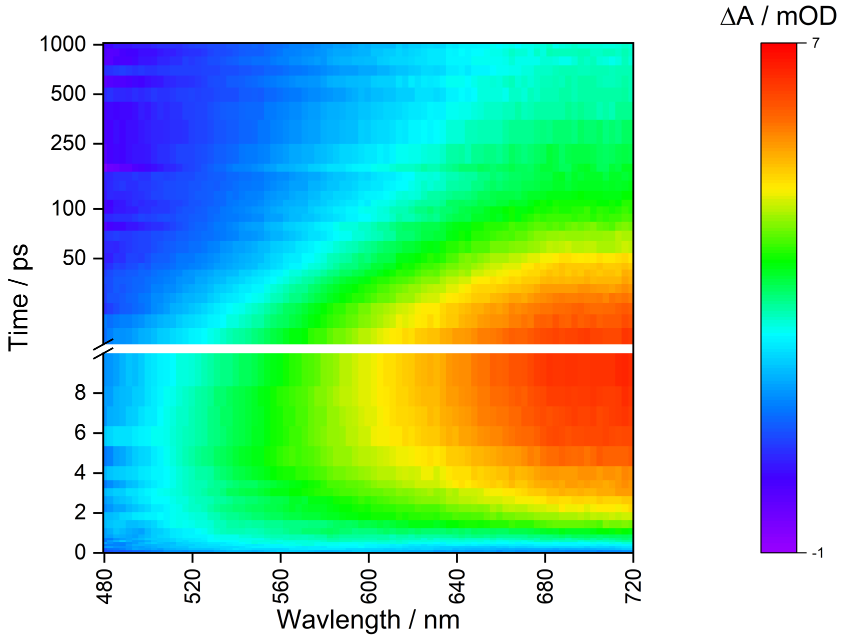

Figure S6: Heat-map of the full 2D transient absorption data set of 20 mM phenolate following photoexcitation at 275 nm. The time axis has a linear scale from 0-10 ps and a logarithmic from 10-1000 ps.

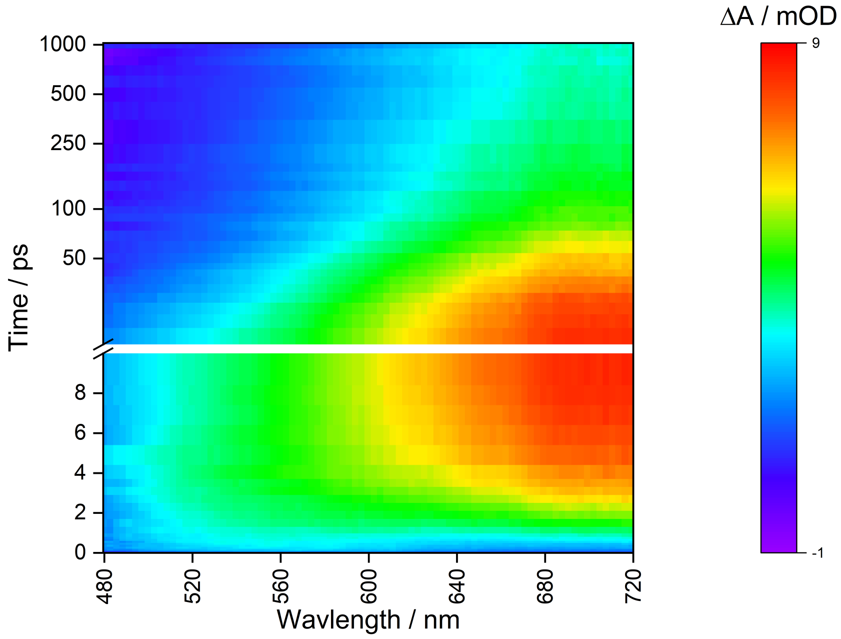

Figure S7: Heat-map of the full 2D transient absorption data set of 20 mM phenolate following photoexcitation at 287 nm. The time axis has a linear scale from 0-10 ps and a logarithmic from 10-1000 ps.

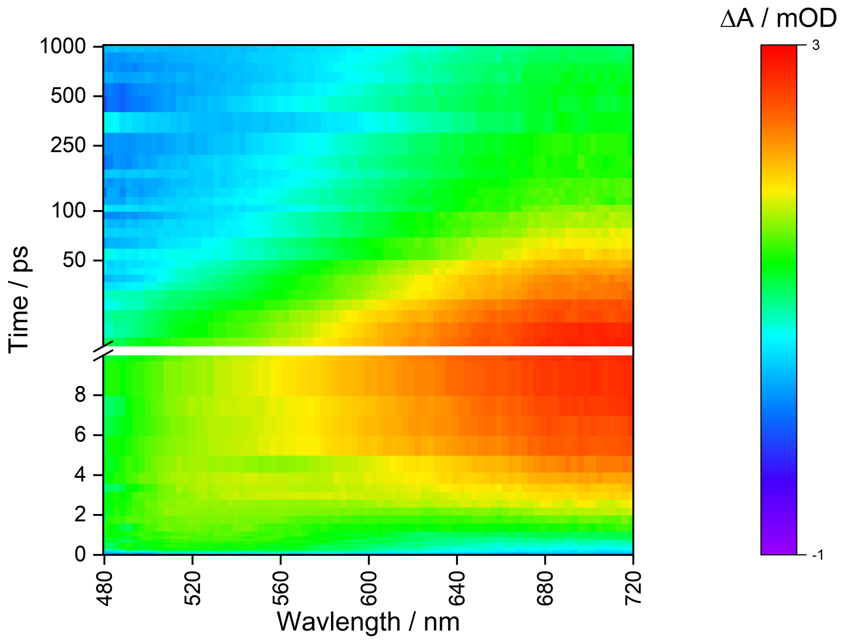

Figure S8: Heat-map of the full 2D transient absorption data set of 20 mM phenolate following photoexcitation at 300 nm. The time axis has a linear scale from 0-10 ps and a logarithmic from 10-1000 ps.

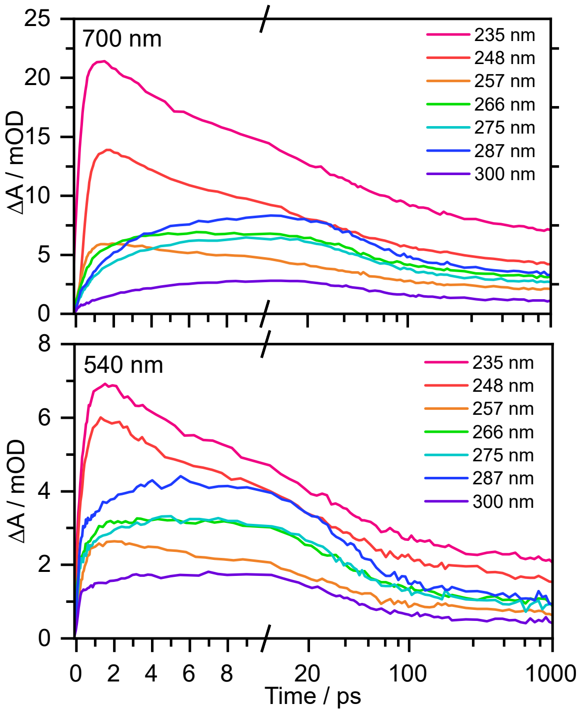

Figure S9: Kinetic traces of the transient absorption spectra of 20 mM aqueous phenolate at a probe wavelength of 700 nm (top) and 540 nm (bottom) and displayed pump wavelengths.

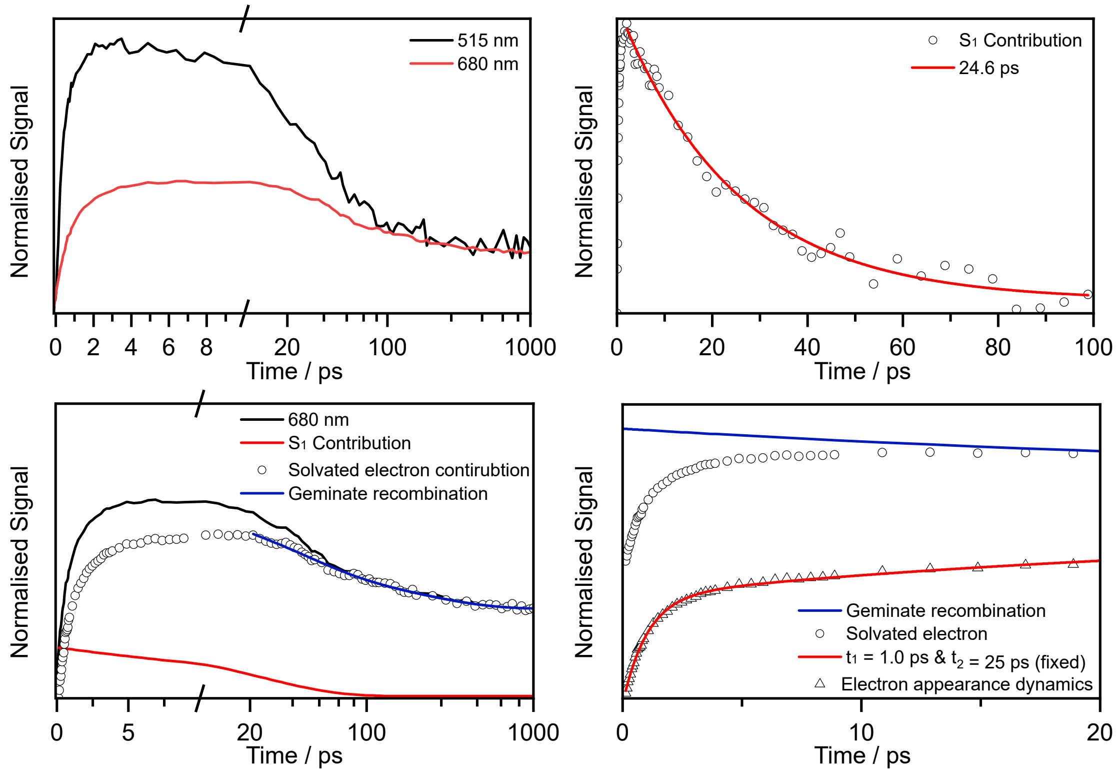

Figure S10: Data analysis procedure for a pump wavelength of 266 nm. Top left displays kinetic traces at probe wavelengths 515 nm and 680 nm. 515 nm is used to isolate the signal from S_1 and 680 nm for the solvated electron. The data is then normalised to 500 ps as there will only be a contribution from the solvated electron signal at this long time. The difference in the contribution is then plotted in the top right figure. A single exponential decay is fitted to the first 100 ps to give the S_1 lifetime, 25 ps. The bottom left figure displays the S_1 contribution (red) with its instantaneous amplitude set to match that of the transient absorption at 680 nm (black). This S_1 contribution is subtracted from the absorption at 680 nm to isolate the contribution from the solvated electron. The solvated electron (circles) signal is fitted (blue) from 20 ps to 1000 ps to extract time constants for geminate recombination. This is reasonably described by a biexponential decay although it is clear the signal is long lived beyond the experimental window. The bottom right plot then fits the dynamics of the electron signal appearing: the geminate recombination (blue line) is interpolated to early times and is subtracted from the total electron signal (circles) to isolate the appearance dynamics (triangles). The precursor states of the electron signal are hot and cold S_1 so the lifetime of S_1 is fixed and a biexponential is fitted. The formation time from hot S_1 is then extracted to be 1.0 ps.

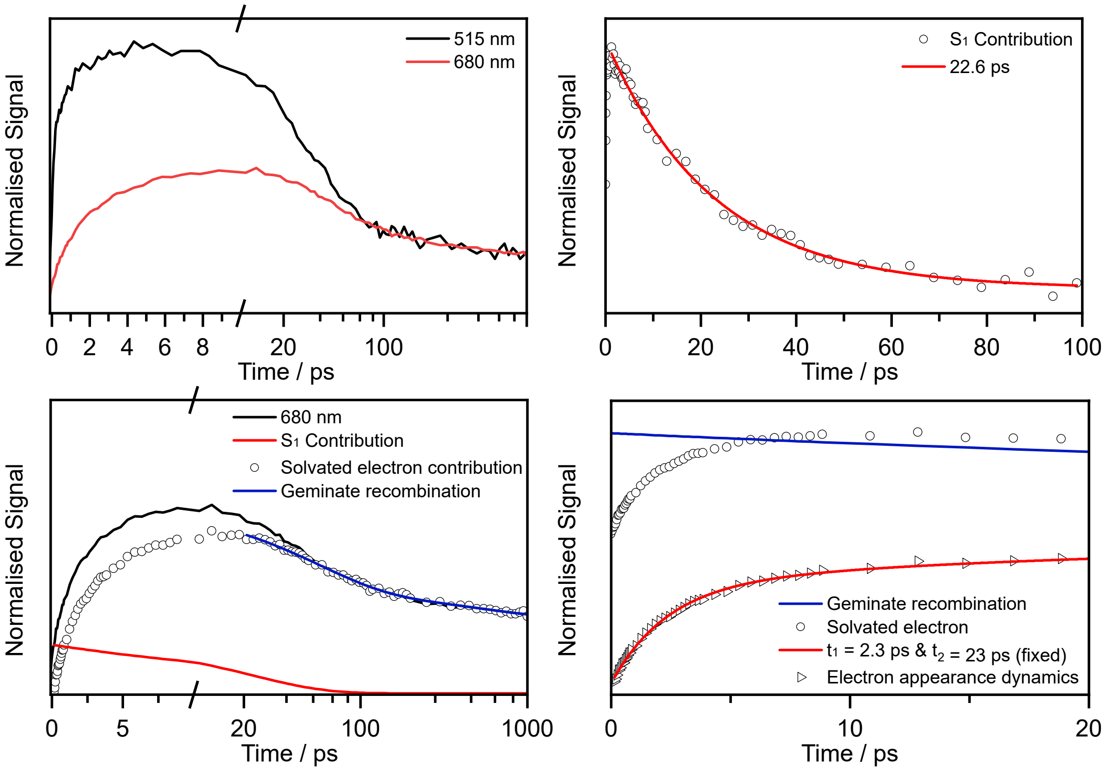

Figure S11: Data analysis procedure for a pump wavelength of 275 nm. Top left displays kinetic traces at probe wavelengths 515 nm and 680 nm. 515 nm is used to isolate the signal from S_1 and 680 nm for the solvated electron. The data is then normalised to 500 ps as there will only be a contribution from the solvated electron signal at this long time. The difference in the contribution is then plotted in the top right figure. A single exponential decay is fitted to the first 100 ps to give the S_1 lifetime, 23 ps. The bottom left figure displays the S_1 contribution (red) with its instantaneous amplitude set to match that of the transient absorption at 680 nm (black). This S_1 contribution is subtracted from the absorption at 680 nm to isolate the contribution from the solvated electron. The solvated electron (circles) signal is fitted (blue) from 20 ps to 1000 ps to extract time constants for geminate recombination. This is reasonably described by a biexponential decay although it is clear the signal is long lived beyond the experimental window. The bottom right plot then fits the dynamics of the electron signal appearing: the geminate recombination (blue line) is interpolated to early times and then is subtracted from the electron signal (circles) to isolate the appearance dynamics of the electron (triangles). The precursor states of the electron signal are hot and cold S_1 so the lifetime of S_1 is fixed and a biexponential is fitted. The formation time from hot S_1 is then extracted to be 2.3 ps.

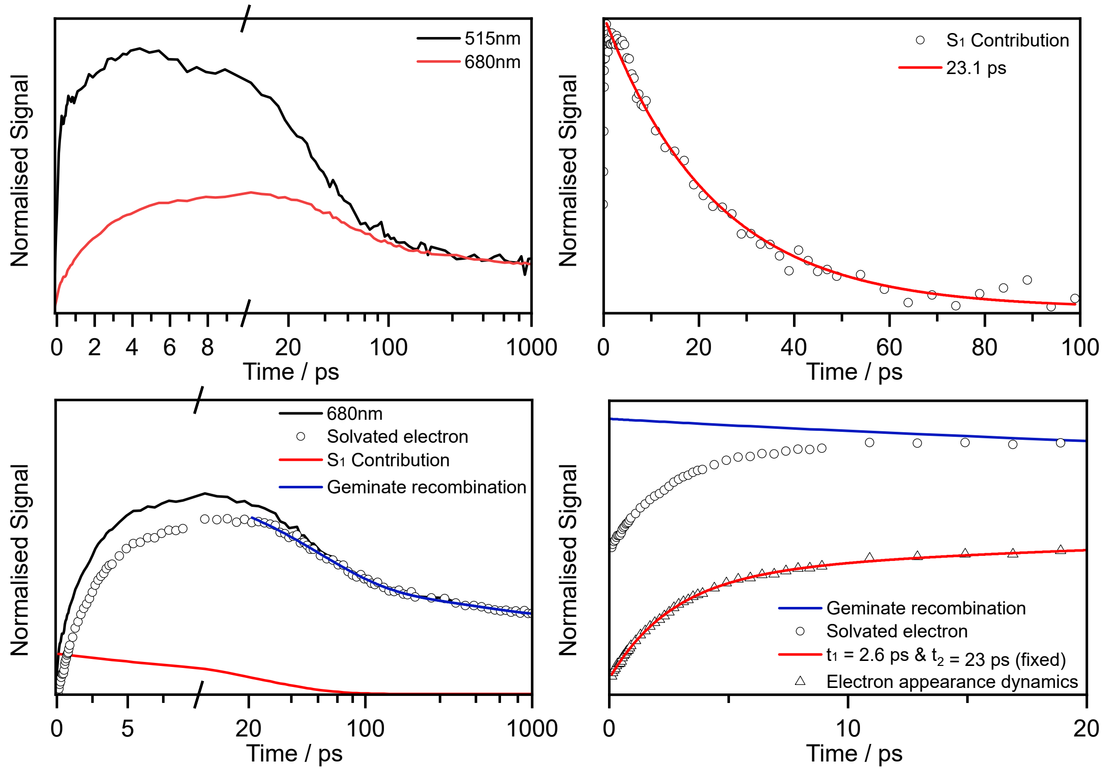

Figure S12: Data analysis procedure for a pump wavelength of 287 nm. Top left displays kinetic traces at probe wavelengths 515 nm and 680 nm. 515 nm is used to isolate the signal from S_1 and 680 nm for the solvated electron. The data is then normalised to 500 ps as there will only be a contribution from the solvated electron signal at this long time. The difference in contribution is then plotted in the top right figure. A single exponential decay is fitted to the first 100 ps to give the S_1 lifetime, 23 ps. The bottom left figure displays the S_1 contribution (red) with its instantaneous amplitude set to match that of the transient absorption at 680 nm (black). This S_1 contribution is subtracted from the absorption at 680 nm to isolate the contribution from the solvated electron. The solvated electron (circles) signal is fitted (blue) from 20 ps to 1000 ps to extract time constants for geminate recombination. This is reasonably describe by a biexponential decay although it is clear the signal is long lived beyond the experimental window. The bottom right plot then fits the dynamics of the electron signal appearing: the geminate recombination (blue line) is interpolated to early times and then subtracted from the electron signal (circles) to isolate the appearance dynamics of the electron (triangles). The precursor states of the electron signal are hot and cold S_1 so the lifetime of S_1 is fixed and a biexponential is fitted. The formation time from hot S_1 is then extracted to be 2.6 ps.

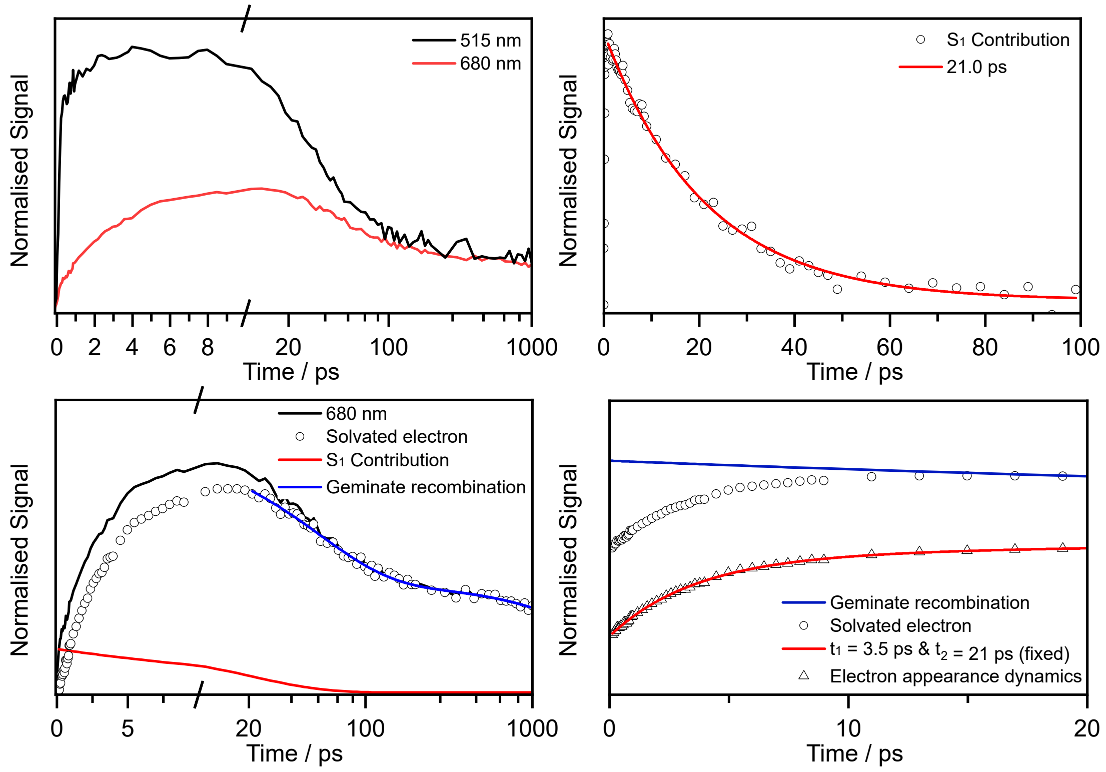

Figure S13: Data analysis procedure for a pump wavelength of 300 nm. Top left displays kinetic traces at probe wavelengths 515 nm and 680 nm. 515 nm is used to isolate the signal from S_1 and 680 nm for the solvated electron. The data is then normalised to 500 ps as there will only be a contribution from the solvated electron signal at this long time. The difference in contribution is then plotted in the top right figure. A single exponential decay is fitted to the first 100 ps and the S_1 lifetime is provided, 21 ps. The bottom left figure displays the S_1 contribution (red) with its instantaneous amplitude is set to match that of the transient absorption at 680 nm (black). This S_1 contribution is subtracted from the absorption at 680 nm to isolate the contribution from the solvated electron. The solvated electron (circles) signal is fitted (blue) from 20 ps to 1000 ps to extract time constants for geminate recombination. This has been fitted with a biexponential, although the error associated with the longer time constant is large suggesting the recombination behaviour could be different for electrons generated with these lower kinetic energies. The longer time constant obtained from this fit, 13000 $\pm$ 200000 ps, is longer than the measurement window of our experiment, which could be the source of the large error. The bottom right plot then fits the dynamics of the electron signal appearing: the geminate recombination (blue line) is interpolated to early times and then is subtracted from the electron signal (circles) to isolate the appearance dynamics of the electron (triangles). The precursor states of the electron signal are hot and cold S_1 so the lifetime of S_1 is fixed and a biexponential is fitted. The formation time from hot S_1 is then extracted to be 3.5 ps.

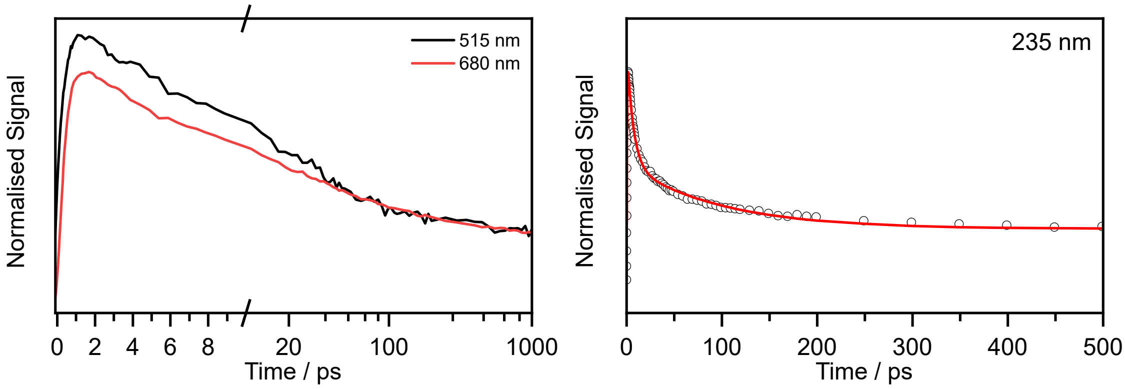

Figure S14: Left: Data displayed is for a pump wavelength of 235 nm and probe wavelengths of 515 nm and 680 nm. Kinetic traces are normalised to 500 ps. As the dynamics at both probe wavelengths are so similar, the contribution from S_1 is minor and can be excluded from the fitting. Right: Four exponential functions convoluted with the IRF. The exponents have lifetimes: 0.36 ps (rise), 6.6 ps (decay), 99 ps (decay) and ∞ (decay). The infinite lifetime is included to account for the solvated electron signal beyond our experimental window.

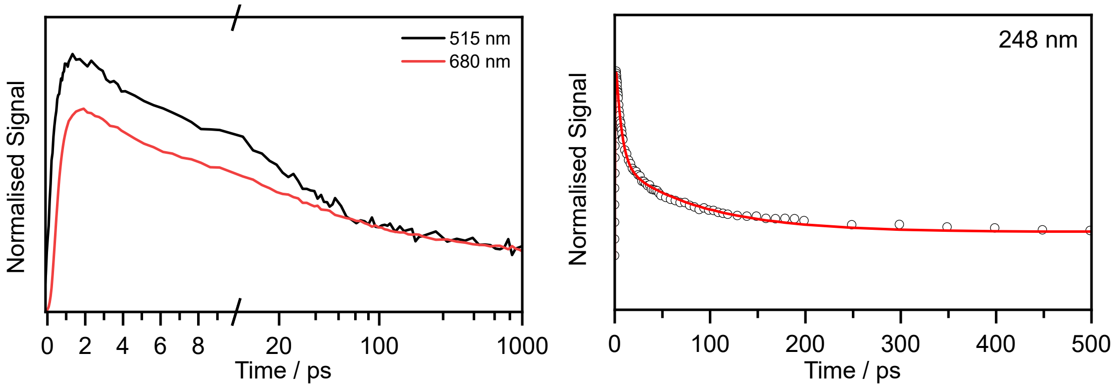

Figure S15: Left: Data displayed is for a pump wavelength of 248 nm and probe wavelengths of 515 nm and 680 nm. Kinetic traces are normalised to 500 ps. The dynamics at both probe wavelengths are very similar, and although there is clearly more contribution from S_1 at this wavelength compared to 235 nm (above), it is still minor and can be excluded from the fitting. Right: Four exponential functions convoluted with the IRF. The exponents have lifetimes: 0.42 ps (rise), 6.2 ps (decay), 90 ps (decay) and ∞ (decay). The infinite lifetime included to account for the solvated electron signal beyond our experimental window.

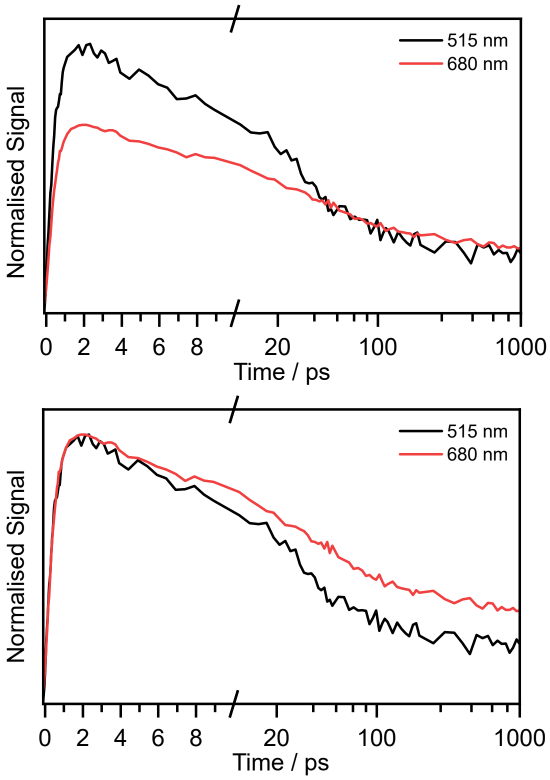

Figure S16: Data displayed is for a pump wavelength of 257 nm and probing wavelengths 515 nm (black) and 680 nm (red). Top: Kinetic traces are normalised to 500 ps. Bottom: Kinetic traces are normalised to the maximum. These plots illustrate the dynamics at probe wavelength 515 nm differ from the dynamics at 680 nm. This would suggest there is a significant contribution from S_1 present. The fast rise time at 680 nm suggests that there is a significant contribution from S_2 (Figs S5 and S6). Therefore, we conclude that at 257 nm both S_1 and S_2 are photoexcited.

S2 Photoelectron spectroscopy

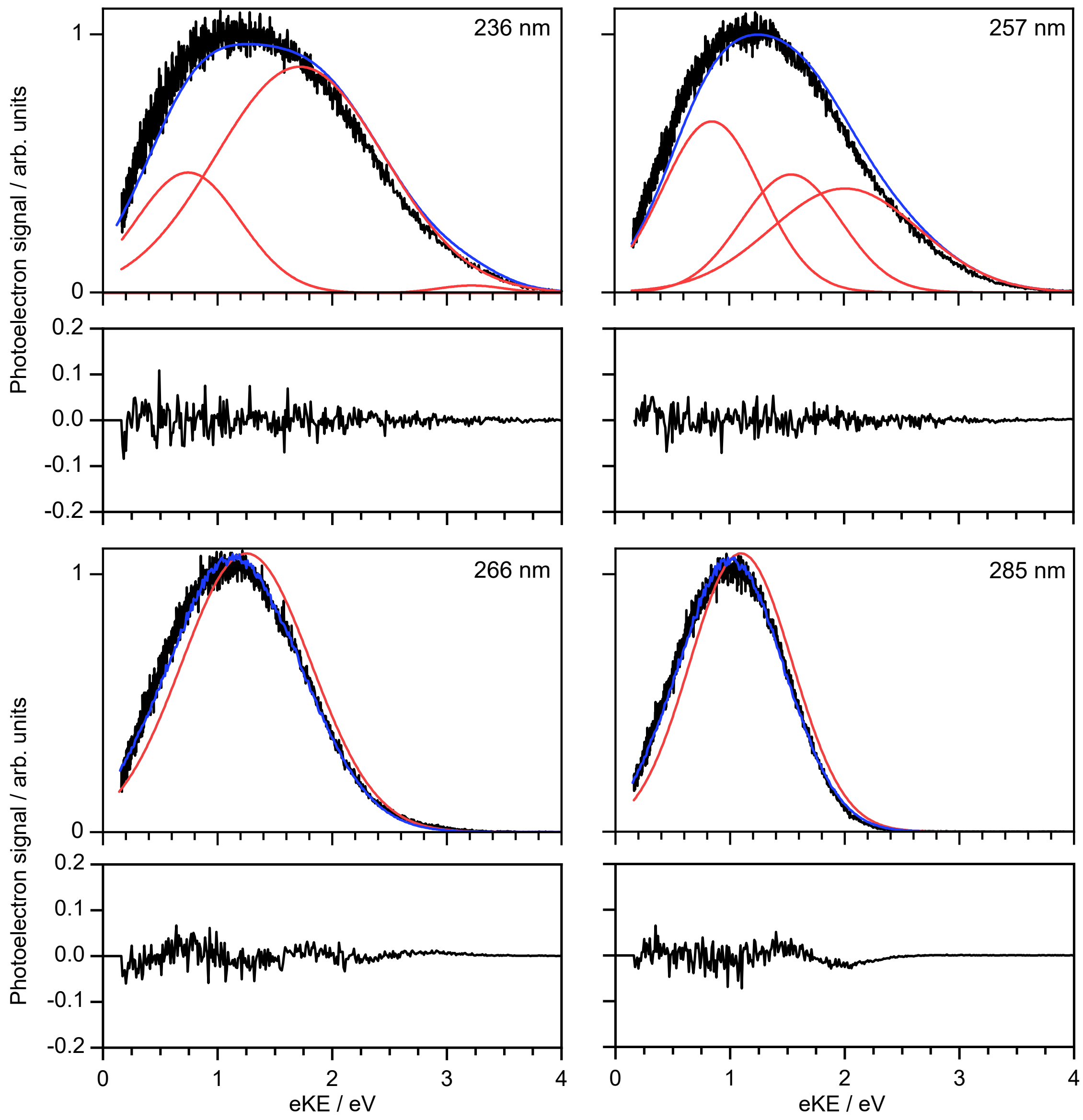

Figure S17: Photoelectron spectra as presented in the body of the paper with raw photoelectron spectra (black), cumulative spectra (dark blue) and decomposed retrieved spectra (red) to indicate detachment pathways present. Residual plots for photoelectron spectra are below the respective plots.

Wavelength / nm (eV)	eKE / eV	FWHM / eV
285.0 (4.35)	1.06±0.07	1.06
266.0 (4.66)	1.25±0.07	1.33
257.0 (4.82)	0.85±0.07	1.01
	1.54±0.07	1.02
	2.01±0.07	1.53
236.0 (5.25)	0.72±0.07	1.08
	1.72±0.07	1.72
	3.22±0.07	0.75

Table S1: Measured centres of Gaussians fitted to the photoelectron spectra of aqueous phenolate corrected for scattering, full-width at half-maximum (FWHM) for each fitted Gaussian, one- and two-photon binding energies. Error bars represent approximate experimental uncertainties.
